# The ameliorating effects of long-term electroacupuncture on cardiovascular remodeling in spontaneously hypertensive rats

**DOI:** 10.1186/1472-6882-14-118

**Published:** 2014-04-01

**Authors:** Ze-Jun Huo, Quan Li, Gui-Hua Tian, Chang-Man Zhou, Xiao-Hong Wei, Chun-Shui Pan, Lei Yang, Yan Bai, You-Yi Zhang, Ke He, Chuan-She Wang, Zhi-Gang Li, Jing-Yan Han

**Affiliations:** 1 Department of Traditional Chinese Medicine, Peking University Third Hospital, Beijing, China; 2 Tasly Microcirculation Research Center, Peking University Health Science Center, Beijing, China; 3 School of Acupuncture and Moxibustion, Beijing University of Chinese Medicine, 11 Bei San Huan Dong Road, Chaoyang District, Beijing 100029, People’s Republic of China; 4 Department of Anatomy, School of Basic Medical Sciences, Peking University, Beijing, China; 5 Institute of Vascular Medicine, Peking University Third Hospital and Key Laboratory of Molecular Cardiovascular Science, Chinese Ministry of Education, Beijing, China; 6 Department of Integration of Chinese and Western Medicine, School of Basic Medical Sciences, Peking University, 38 Xueyuan Road, Beijing 100191, People’s Republic of China

**Keywords:** Cardiac hypertrophy, Electroacupuncture, Angiotensin II type 1 receptor, Endothelin-1, Endothelin-1 type A receptor, NO

## Abstract

**Background:**

The purpose of this study was to investigate the inhibitory effects of long-term electroacupuncture at BaiHui (DU20) and ZuSanLi (ST36) on cardiovascular remodeling in spontaneously hypertensive rats (SHR) and underlying mechanisms.

**Methods:**

6-weeks-old SHR or Wistar male rats were randomly, divided into 6 groups: the control group (SHR/Wistar), the non-acupoint electroacupuncture stimulation group (SHR-NAP/Wistar-NAP) and the electroacupuncture stimulation at DU20 and ST36 group (SHR-AP/Wistar-AP), 24 rats in each group. Rats were treated with or without electroacupuncture at DU20 and ST36, once every other day for a period of 8 weeks. The mean arterial pressure (MAP) was measured once every 2 weeks. By the end of the 8th week, the left ventricular structure and function were assessed by echocardiography. The content of angiotensin II (Ang II), endothelin-1 (ET-1) and nitric oxide (NO) in the plasma was determined using enzyme-linked immunosorbent assay. Histological studies on the heart and the ascending aorta were performed. The expression of angiotensin II type 1 receptor (AT1R), endothelin-1 type A receptor (ETAR), eNOS and iNOS in rat myocardium and ascending aorta was investigated by Western blotting.

**Results:**

The MAP in SHR increased linearly over the observation period and significantly reduced following electroacupuncture as compared with sham control SHR rats, while no difference in MAP was observed in Wistar rats between electroacupuncture and sham control. The aortic wall thickness, cardiac hypertrophy and increased collagen level in SHR were attenuated by long term electroacupuncture. The content of Ang II, ET-1 in the plasma decreased, but the content of NO increased after electroacupuncture stimulation in SHR. Long term electroacupuncture significantly inhibited the expression of AT1R, ETAR and iNOS, whereas increased eNOS expression, in myocardium and ascending aorta of SHR.

**Conclusions:**

The long term electroacupuncture stimulation at DU20 and ST36 relieves the increased MAP and cardiovascular abnormality in both structure and function in SHR, this beneficial action is most likely mediated via modulation of AT1R-AT1R-ET-1-ETAR and NOS/NO pathway.

## Background

Arterial hypertension, a major health problem for its high incidence and concomitant risks of the cardiovascular system, has been identified as the leading risk factor for cardiac mortality [1]. Cardiac remodeling, including ventricular hypertrophy and the subsequent cardiac fibrosis, is the common outcome of human essential hypertension [2]. The resultant cardiac malfunction leads to poor clinical prognosis and ultimately cardiovascular-related death. Accumulating evidence from clinical and experimental studies indicates that angiotensin II (Ang II)/angiotensin II type 1 receptor (AT1R) and endothelin (ET)-1/ endothelin-1 type A receptor (ETAR) have important roles in hypertension and pathological remodeling of the heart [3,4]. Intriguingly, Ang II also induces ET-1 via ERK and reactive oxygen species, suggesting that ET operates downstream of the Ang II system to drive fibroblast activation and fibrosis [5]. Dual-acting Ang II and ET receptor blockers have been shown to reduce systemic blood pressure in animal models and in hypertensive patients. Thus, Ang II/ET combination therapies have promise in controlling cardiac disease [6]. In addition, nitric oxide (NO) produced in the heart by nitric oxide synthase (NOS) is a highly reactive signaling molecule and an important modulator of cardiac function. In the heart, endothelial NOS (eNOS) is constitutively present in distinct subcellular locations within cardiomyocytes, whereas inducible NOS (iNOS) is absent in the healthy heart, but is induced by pro-inflammatory mediators [7]. In eNOS^−/−^ mice pressure overload induces more severe left ventricular (LV) hypertrophy, LV dysfunction, and cardiac fibrosis than in wild-type mice [8]. But iNOS-deficient mice subjected to aortic constriction demonstrates much less hypertrophy, dilatation, fibrosis, and dysfunction of the LV than wild-type mice with aortic constriction [9].

Electroacupuncture, a traditional therapy, has been recommended as a complementary therapy for hypertension. Baihui (DU20), alone or in combination with other acupuncture points, has been used for the treatment of hypertension and prehypertension patients [10]. Zusanli (ST36) is known as an acupoint for its role in reducing blood pressure [11]. Previous research has proved that the acupuncture at ST-36 could reduce the level of Ang II and ET-1 in peripheral blood [12], increase the level of NO and adjust the expression of eNOS or iNOS [11,13]. However, it is so far unclear whether or not electroacupuncture at both acupoints DU20 and ST36 can prevent the cardiovascular remodeling secondary to hypertension and, if yes, what are the underlying mechanisms.

In the present study, using spontaneously hypertensive rats (SHR), we demonstrated the recovery effects of long-term electroacupuncture at DU20 and ST36 on cardiovascular remodeling, which may be related to the modulation of Ang II, ET-1 and NO, and expression of angiotensin II type 1 receptor (ATIR), endothelin-1 type A receptor (ETAR), eNOS and iNOS in heart and aortic tissues.

## Methods

### Experimental animal

Male SHR and Wistar rats, 6 weeks old, weighing 120–130 g, were purchased from the Animal Center of Peking University Health Science Center (Certificate NO. SCXK 2002–0001). The rats were maintained in a quiet room at constant temperature (20-22°C) and humidity (50-60%) with 12 h light–dark cycle. All animals were handled according to the guidelines of the Peking University Health Science Center Animal Research Committee. The investigations conformed to the Guide for the Care and Use of Laboratory Animals (NIH publication no. 85–23, 1996), and were approved by Peking University Biomedical Ethics Committee Experimental Animal Ethics Branch (LA2011-38).

### Animal grouping and electroacupuncture stimulation

The rats were randomly divided into 6 groups, the control group (SHR/Wistar), the non-acupoint electroacupuncture stimulation group (SHR-NAP/Wistar-NAP) and the electroacupuncture stimulation at DU20 and ST36 group (SHR-AP/Wistar-AP), with 24 rats in each group. The number of animals for determination of each parameter in various group is detailed in Table 1.

**Table 1:** The number of animals for different experimental groups and various parameters

	**Control (SHR/Wistar)**	**Non-acupoint electroacupuncture stimulation group (SHR-NAP/Wistar-NAP)**	**Electroacupuncture stimulation at DU20 and ST36 group (SHR-AP/Wistar-AP)**	**Total**
Echocardiographic analysis	6/6	6/6	6/6	36
Mean arterial pressure and heart to body weight ratio	8/8	8/8	8/8	48
HE staining	3/3	3/3	3/3	18
Sirius red staining	3/3	3/3	3/3	18
ELISA and Western Blotting	4/4	4/4	4/4	24
Total	24/24	24/24	24/24	144

The mean arterial pressure was conducted once every 2 weeks. Eight weeks after electroacupuncture stimulation, echocardiographic analysis, assay of heart to body weight ratio, ELISA, HE and Sirius red staining, and Western blotting were conducted in sequence.

For the SHR-AP and Wistar-AP groups, the animals were subjected to stimulation by electroacupuncture at acupoint DU20 (located on the top of the head at the intersection of middle sagittal line and the connection of two ear apexes) and ST36 (5 mm below head of right fibula under knee joint, and 2 mm lateral to the anterior tubercle of the tibia). Sterilized disposable stainless steel needles (0.3 mm × 40 mm, Global brand, Suzhou, China) were inserted 2 mm deep at DU20 with a slope of 30 degrees. Perpendicular needling was performed with the depth of 5 mm at ST36. Both needles were connected to Han’s Acupoint Nerve Stimulator (Model LH 202H, Huawei Ltd, Beijing, China). To keep animals quiet during electro-stimulation, the rats were fastened to an animal plate and adapted for 10 min before electroacupuncture stimulation. Electric stimulation proceeded for 20 minutes each time, once every other day, for a period of 8 weeks, and the stimulation parameters were set at disperse-dense waves of 2/100 Hz with an intensity of 1 mA, 2 Hz [14]. For the SHR-NAP and Wistar-NAP groups, the animals received similar treatment as electroacupuncture groups but the electroacupuncture site was 1 cm and 2 cm from the root of the tail, respectively, to replace DU20 and ST36. The animals in the control groups were fastened to an animal plate without acupuncture and electrical stimulation.

### Measurement of mean arterial pressure (MAP) and heart to body weight ratio

Blood pressure was measured as described [15] with modification. The measurement was conducted once every 2 weeks at 8 am in a quiet room. After staying in a box at 29 ± 1°C for 10 min, the blood pressure was measured with a blood pressure monitor (BP-98A, Softron, Tokyo, Japan), taking the average of three consecutive measurements as the mean arterial pressure (MAP). The rats were killed 8 weeks after electroacupuncture stimulation, and the heart to body weight ratio (HW/BW) was calculated.

### Echocardiographic analysis

The left ventricle function was evaluated 8 weeks after electroacupuncture stimulation using a Vevo 770 High-Resolution Imaging Systems (Visual Sonics, Toronto, Canada) with a 17.5 MHz linear array transducer (model 716). Briefly, rats were anaesthetized with 1.5-2.0% isoflurane by mask, the chest was shaved, the animal situated in the supine position on a warming pad, and electrocardiogram limb electrodes were placed. Two-dimensional cine loops and guided M-mode frames were recorded from the parasternal short and long axis. All data were analyzed off-line at the end of the study with software resident on the ultrasound system. The following parameters were measured as indicators of function and remodeling: left ventricular anterior wall (LVAW), left ventricular posterior wall (LVPW), left ventricular internal diameter (LVID), left ventricle ejection fraction (%EF), and left ventricle fractional shortening (%FS) [16].

### Histological evaluation of ascending aorta

Ascending aorta was removed after 8 weeks of electroacupuncture stimulation, fixed in 4% formaldehyde, and further prepared for paraffin sectioning [17]. The sections (5 μm) were deparaffinized and rehydrated, and stained with hematoxylin and eosin. The images were captured by a digital camera connected to a microscope (BX512DP70, Olympus, Tokyo, Japan).

### Myocardial collagen stain

Myocardial collagen content was determined using the sirius red staining technique [18]. Briefly, hearts were perfusion-fixed 8 weeks after electroacupuncture stimulation, processed for paraffin section and stained with picrosirius red. The images were captured by a digital camera connected to a microscope (BX512DP70, Olympus, Tokyo, Japan).

### Determination of Ang II, ET-1 and NO in the plasma

The rats were anesthetized with urethane (2.0 g/kg body weight, i.p.), blood samples (2 mL) were collected from the carotid arteries into the tube containing EDTA and centrifuged (1000 × g, 20 min). The content of Ang II, ET-1 and NO in the plasma was determined by ELISA following manufacture’s protocol (R&D, Minneapolis, MN, USA).

### Western blotting

Heart and aorta were removed 8 weeks after electroacupuncture stimulation. The tissues were lysed in RIPA buffer containing phosphatase and protease inhibitors and the protein content was quantified with a Bio-Rad kit (Bio-Rad, Hercules, CA, USA) following the conditions suggested by the manufacturer. Equal amount of total protein was subjected to SDS-PAGE and blotted. The PVDF Membranes (Millipore, Billerica, MA, USA) were incubated with specific primary antibodies overnight at 4°C followed by HRP-labeled secondary antibodies for 1 h at room temperature. The primary antibodies used included AT1R antibody (1:300), ETAR antibody (1:300), eNOS antibody (1:1000) and iNOS anibody (1:1000) (Abcam, Cambridge, MA, USA). The targeted proteins were detected by using enhanced chemiluminescence system (Pierce,Rockford,IL, USA). Densitometric analyses of Western blots were performed using the Quantity One image analyzer software (Bio-Rad, Hercules, CA, USA). The analysis was performed using volume rectangular tool. For background subtraction the same area was selected above and below the bands using the global method for background subtraction [19].

### Statistical analysis

All data were expressed as mean ± SEM. For comparison of >2 conditions a one-way analysis of variance (ANOVA) with Turkey post hoc test or a repeated measures ANOVA with Bonferroni post hoc test was used. A probability of less than 0.05 was considered to be statistically significant.

## Results

### Long term electroacupuncture reduces the MAP in SHR

**Figure 1:**
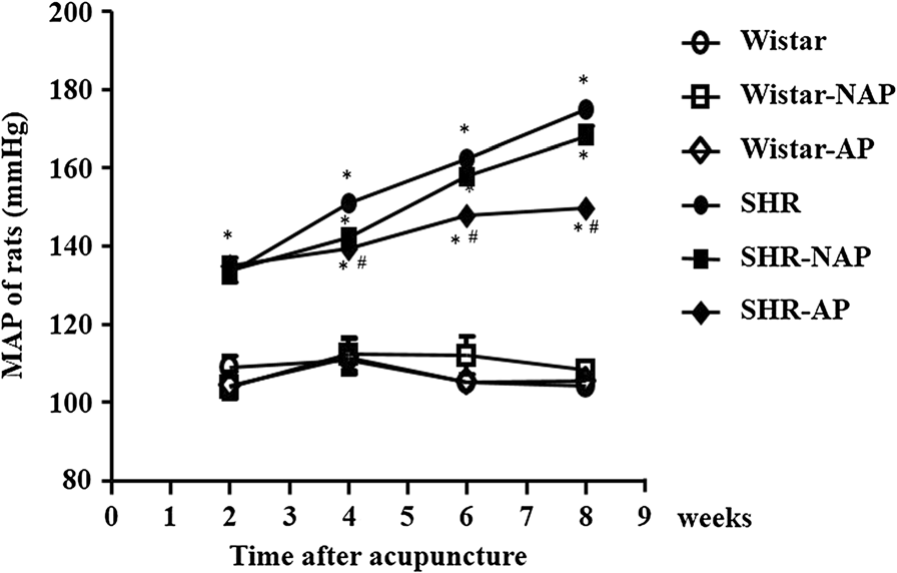
**The effect of electroacupuncture on rat means arterial pressure.** For each group, the mean arterial pressure is measured by tail-cuff method after 2, 4, 6 and 8 weeks of electroacupuncture. Wistar: Wistar control group. Wistar-NAP: Wistar non-acupoint group. Wistar-AP: Wistar DU20 and ST36 acupoints group. SHR: SHR control group. SHR-NAP: SHR non-acupoint group. SHR-AP: SHR DU20 and ST36 acupoints group. Values are means ± SEM from 8 animals. *p < 0.05 *vs.* Wistar group, ^#^*p* < 0.05 *vs.* SHR group.

The effect of long-term stimulation with electroacupuncture at DU20 and ST36 on MAP during the observation period is shown in Figure 1. The MAP in Wistar groups remained nearly unchanged during 8 weeks of electroacupuncture stimulation. In contract, MAP in SHR group increased with time, from 130 mmHg at week 2 to 170 mmHg at week 8. MAP in SHR-NAP group changed over time similarly to that in SHR group, and no significant difference was observed at any time point between the two groups. Of notice, MAP in SHR-AP group was attenuated significantly starting from week 4, as compared to SHR group.

### Long term electroacupuncture decreases the wall thickness of left ventricle and improves the heart function

**Figure 2:**
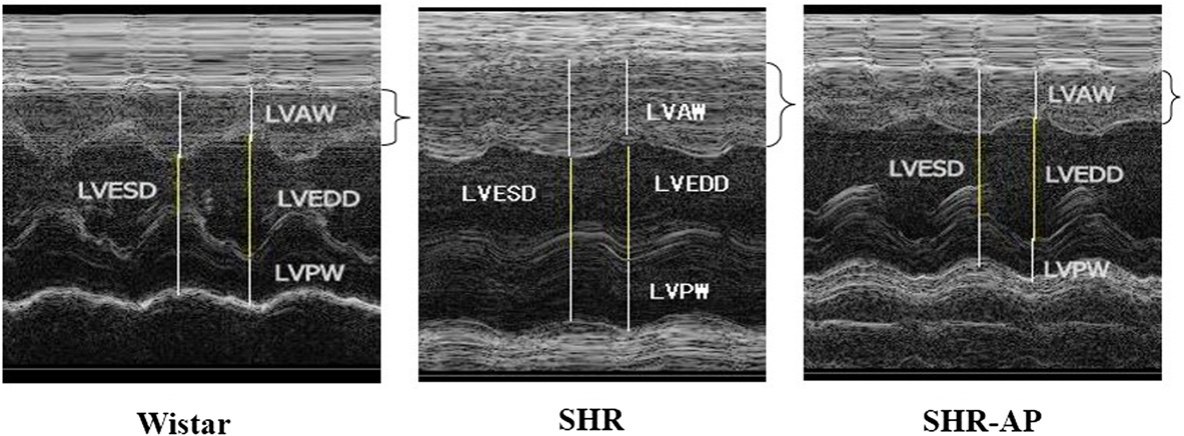
**Representative echocardiograms of rat hearts in various conditions for determination of the ventricle wall thickness (n = 6).** The ventricular anterior wall thickness was shown in brackets. Wistar: Wistar control group. SHR: SHR control group. SHR-AP: SHR DU20 and ST36 acupoint group. LVAW, left ventricular anterior wall.

M-mode echocardiograms of the rat hearts in various conditions were acquired 8 weeks after electroacupuncture stimulation and the representative images are presented in Figure 2. Compared to Wistar rat, the heart of SHR exhibited marked hypertrophy with significantly increased LVAW and LVPW. The hypertrophy was ameliorated by 8 weeks of electroacupuncture stimulation at DU20 and ST36.

**Figure 3:**
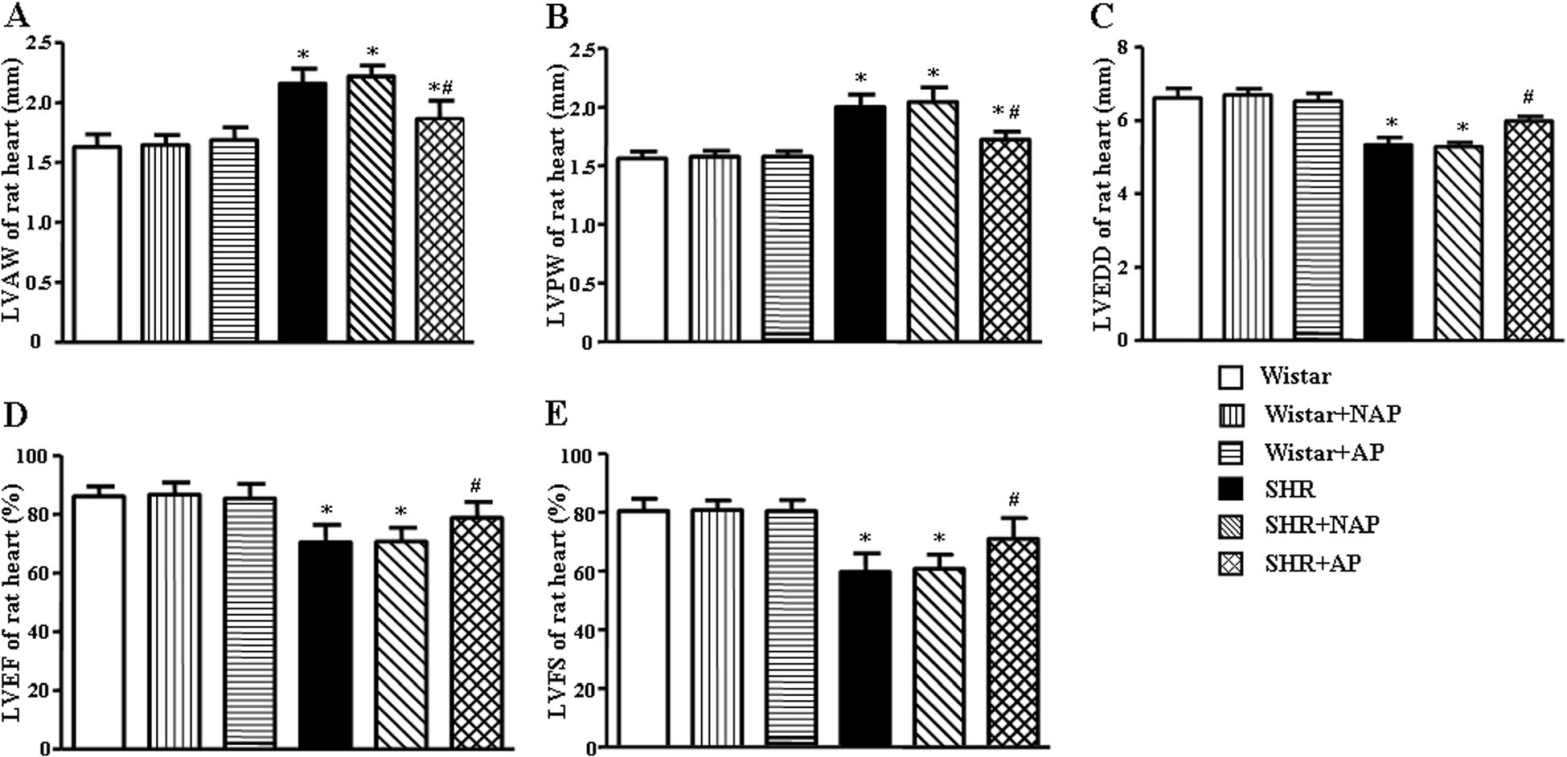
**The effect of electroacupuncture stimulation on parameters of rat cardiac structure and heart function in different conditions.** The parameters were determined 8 weeks after electroacupuncture stimulation. **A**, **B**, **C**, **D** and **E** are the results of statistical analysis for the anterior LVAW, LVID, LVPW, EF value, left ventricular FS, respectively. Wistar: Wistar control group. Wistar-NAP: Wistar non-acupoint group. Wistar-AP: Wistar DU20 and ST36 acupoints group. SHR: SHR control group. SHR-NAP: SHR non-acupoint group. SHR-AP: SHR DU20 and ST36 acupoints group. Values are means ± SEM from 6 animals. *p < 0.05 *vs.* Wistar group, ^#^*p* < 0.05 *vs.* SHR group.

The beneficial action of electroacupuncture stimulation at DU20 and ST36 for heart function of SHR was confirmed by quantitative analysis of the echocardiograms, as shown in Figure 3. Compared with Wistar rat, LVAW and LVPW of SHR significantly increased, whereas LVEF and LVFS markedly decreased, which suggested dilative remodeling and systolic dysfunction. Long term electroacupuncture stimulation at DU20 and ST36 attenuated these impairments significantly.

### Long term electroacupuncture decreases the ratio of HW to BW of SHR

**Figure 4:**
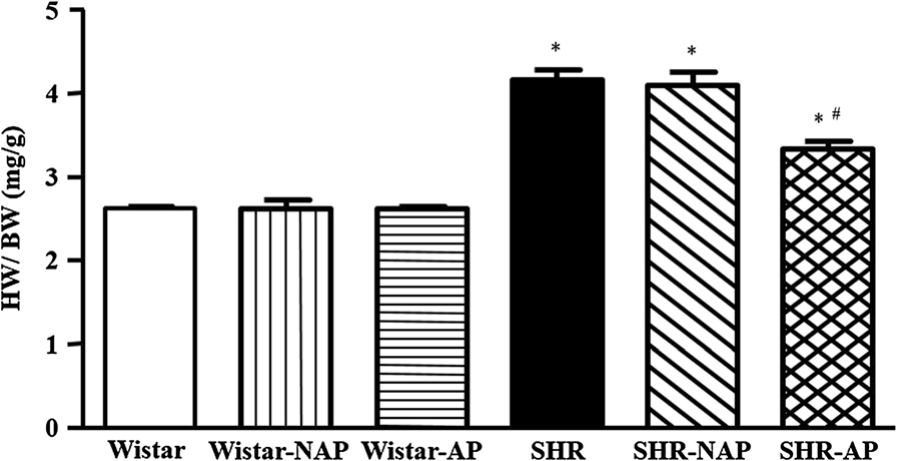
**The effect of electroacupuncture on rat HW/BW.** The HW to BW ratio was estimated 8 weeks after electroacupuncture stimulation in different experimental conditions. Wistar: Wistar control group. Wistar-NAP: Wistar non-acupoint group. Wistar-AP: Wistar DU20 and ST36 acupoints group. SHR: SHR control group. SHR-NAP: SHR non-acupoint group. SHR-AP: SHR DU20 and ST36 acupoints group. Values are means ± SEM from 8 animals. *p < 0.05 *vs.* Wistar group, ^#^*p* < 0.05 *vs.* SHR group.

Heart to body weight ratio was used as a parameter reflecting the size of the entire heart, and the results were presented as graphics in Figure 4. In the SHR controls, the average HW/BW was found to be higher than that of Wistar group. Long term electroacupuncture stimulation at DU20 and ST36 resulted in significant reduction in HW/BW, compared with untreated SHR. However, there was no significant difference between SHR control and SHR-NAP group. No obvious difference in HW/BW was observed either among Wistar group, Wistar-NAP group and Wistar-AP group.

### Long term electroacupuncture reduces the wall thickness in ascending aorta and attenuates cardiac fibrosis in SHR

**Figure 5:**
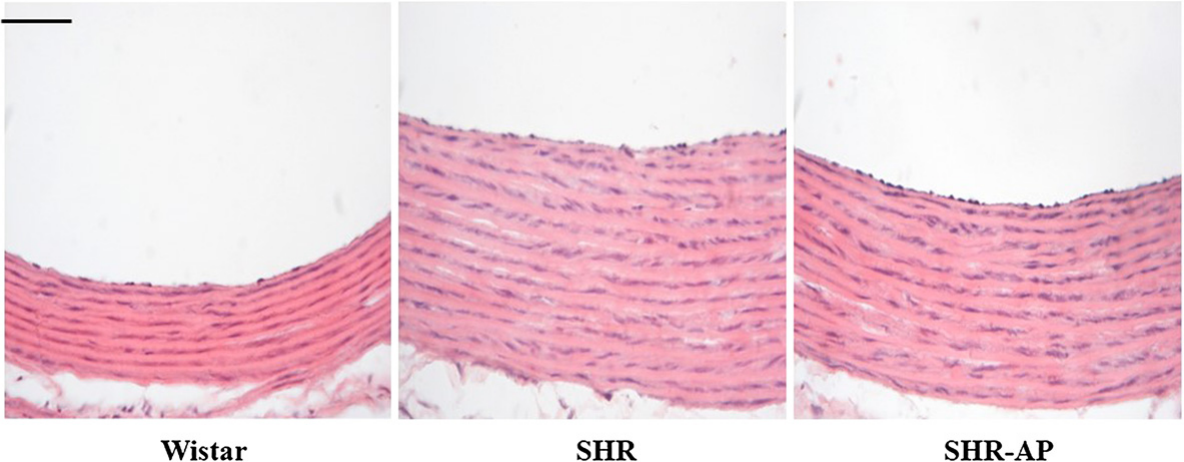
**The effect of electroacupuncture on histology of rat aortic tissue.** The tissue was taken 8 weeks after electroacupuncture stimulation from animals in different conditions and stained by hematoxylin and eosin (n = 3). Wistar: Wistar control group. SHR: SHR control group. SHR-AP: SHR DU20 and ST36 acupoints group. Bar = 50 μm.

Figure 5 illustrates the results of histological examination of the ascending aorta in different groups. Compared with the Wistar group, wall thickness of ascending aorta in SHR markedly increased. Long term electroacupuncture stimulation at DU20 and ST36 reduced the wall thickness of ascending aorta in SHR.

**Figure 6:**
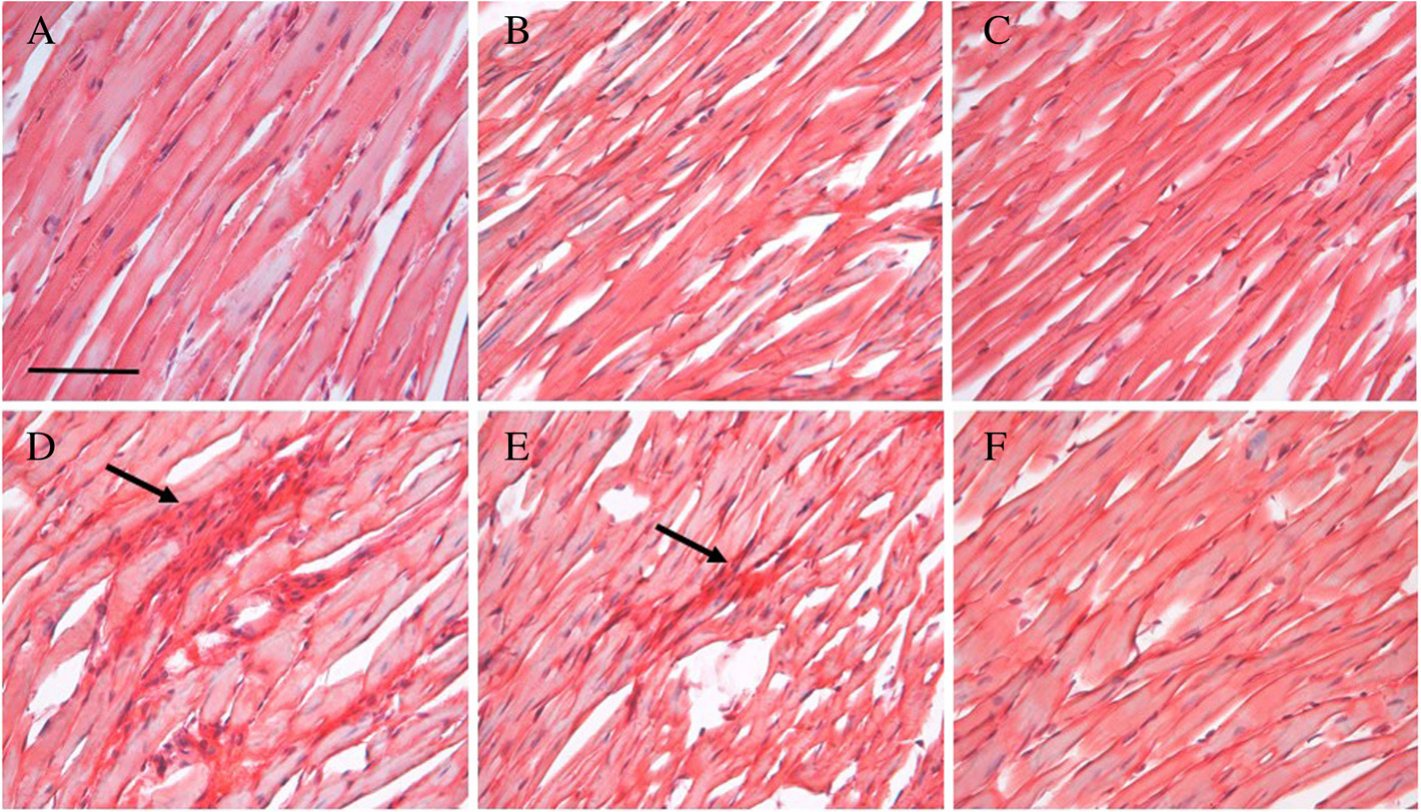
**The effect of electroacupuncture on collagen deposition in rat myocardium.** Collagen was stained by sirius red (n = 3). **A**: Wistar control group. **B**: Wistar non acupoint group. **C**: Wistar DU20 and ST36 acupoints group. **D**: SHR control group. **E**: SHR non acupoint group. **F**: SHR DU20 and ST36 acupoints group. The arrow pointed to the collagen fiber in myocardium. Bar = 50 μm.

Sirius red staining was applied to examine the collagen deposition in myocardium in different conditions 8 weeks after electroacupuncture stimulation, and the result is presented in Figure 6. As evidenced from the representative images, collagen deposition increased significantly in SHR group, compared with Wistar group, and the increase was ameliorated by long term electroacupuncture stimulation at DU20 and ST36.

### Effect of long term electroacupuncture on Ang II, ET-1 and NO production

**Figure 7:**
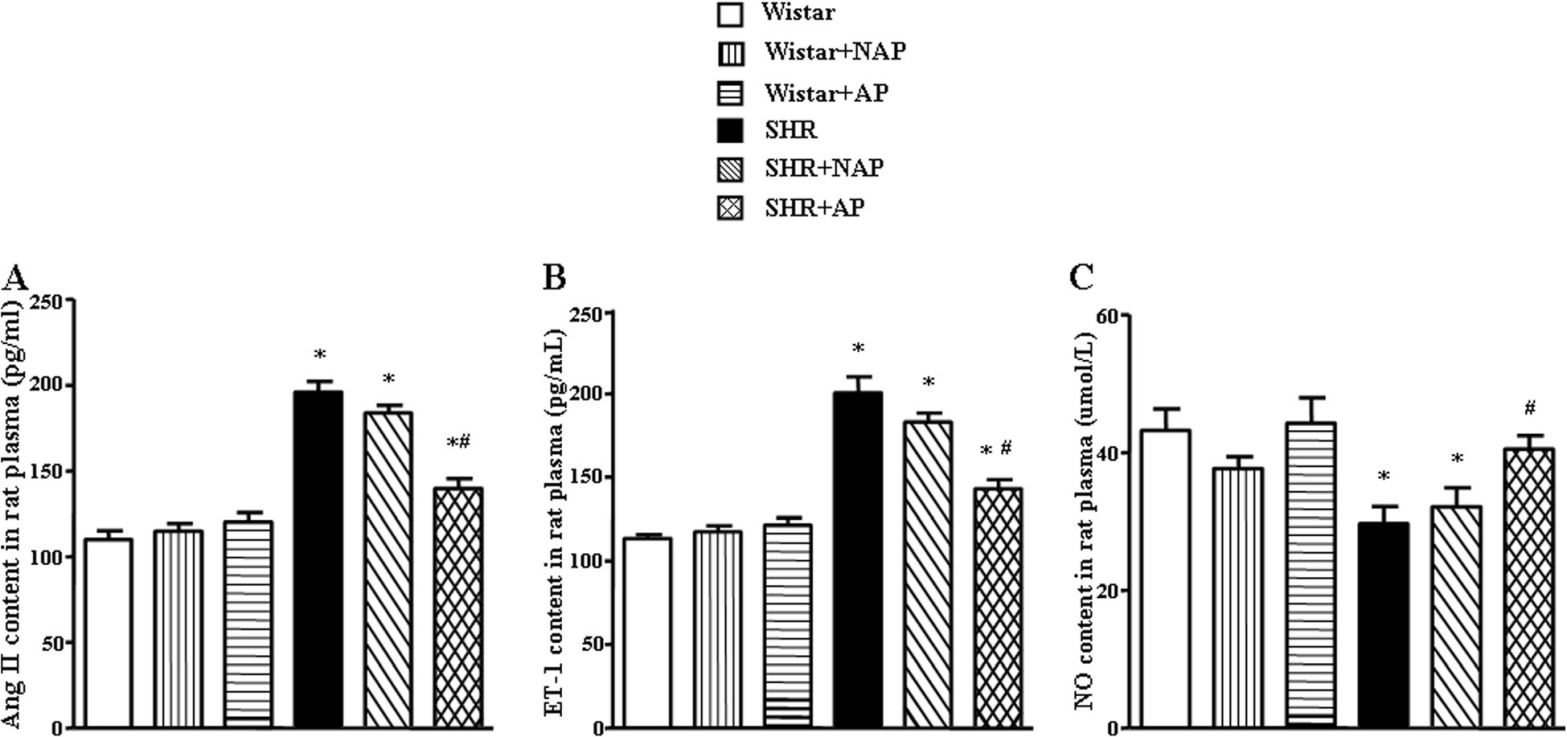
**The effect of electroacupuncture on the content of Ang II, ET-1 and NO in rat plasma. A**, **B** and **C** are the results of statistical analysis for the content of Ang II, ET1 and NO after 8 weeks of electroacupuncture stimulation, respectively. Wistar: Wistar control group. Wistar-NAP: Wistar non-acupoint group. Wistar-AP: Wistar DU20 and ST36 acupoints group. SHR: SHR control group. SHR-NAP: SHR non-acupoint group. SHR-AP: SHR DU20 and ST36 acupoints group. Values are means ± SEM from 4 animals. *p < 0.05 *vs.* Wistar group, ^#^*p* < 0.05 *vs.* SHR group.

The content of Ang II, ET-1 and NO had no distinct difference among Wistar group, Wistar-NAP group and Wistar-AP group (Figure 7). Compared to the Wistar group, the content of Ang II and ET-1 in SHR plasma significantly increased, but the content of NO decreased. Long term electroacupuncture stimulation at DU20 and ST36 could effectively inhibit these changes.

### Effect of long term electroacupuncture on protein expression in aorta and myocardium

**Figure 8:**
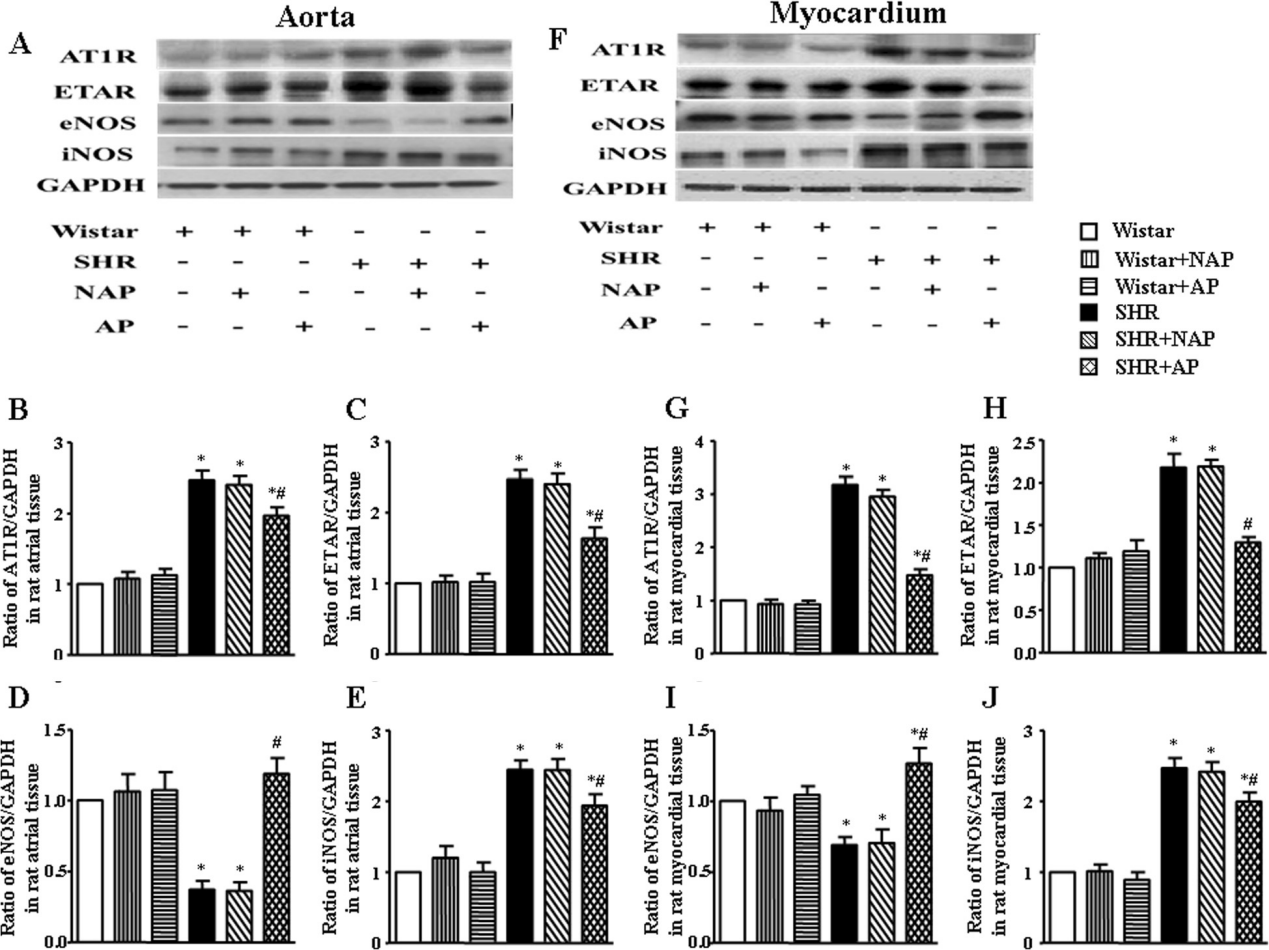
**Western blotting analysis of ATIR, ETAR, eNOS, and iNOS protein in aorta and myocardium, respectively, from Wistar and SHR in different conditions after 8 weeks of electroacupuncture stimulation. A** and **F** show the representative blots of ATIR, ETAR, eNOS, and iNOS protein in aorta and myocardium, respectively. **B** and **G**, **C** and **H**, **D** and **I**, and **E** and **J** show densitometric analysis of the western blot of AT1R, ETAR, eNOS and iNOS protein in aorta and myocardium, respectively. Relative abundance of protein was calculated against GAPDH. Wistar: Wistar control group. Wistar-NAP: Wistar non-acupoint group. Wistar-AP: Wistar DU20 and ST36 acupoints group. SHR: SHR control group. SHR-NAP: SHR non-acupoint group. SHR-AP: SHR DU20 and ST36 acupoints group. Values are means ± SEM from 4 animals. *p < 0.05 *vs.* Wistar group, ^#^*p* < 0.05 *vs.* SHR group.

Figure 8 shows the results of western blotting analysis for ATIR, ETAR, eNOS and iNOS in myocardium and aorta of each group after 8 weeks of electroacupuncture stimulation, respectively. The protein expression of ATIR, ETAR, eNOS and iNOS of aorta or myocardium has no obvious difference among Wistar group, Wistar-NAP group and Wistar-AP group. In contrast, the protein expression of ATIR, ETAR and iNOS in the SHR group was significantly higher than that of age-matched Wistar group, but the eNOS expression decreased significantly. Long term electroacupuncture stimulation at DU20 and ST36 effectively reduced the expression of ATIR, ETAR and iNOS, whereas increased eNOS expression, in aorta and myocardium of SHR.

## Discussion

The present study provided structural and functional evidence for the cardiac remodeling, including hypertrophy and fibrosis, during the chronic stage of hypertension in SHR. Cardiac hypertrophy and fibrosis were significantly reversed by long term electroacupuncture stimulation at DU20 and ST36, as indicated by echocardiography, haematoxylin and eosin staining and sirius red staining. The attenuation of cardiac remodeling in SHR was accompanied by the decreased content of Ang II and ET-1 in the plasma, and decreased expression of ATIR and ETAR in aorta and myocardium in SHR. These results indicated that long term electroacupuncture may favorably modify the development of cardiac remodeling by affecting the local rennin-angiotensin system on the cardiac cells. Furthermore, we found that cardiac remodeling was associated with a decline of NO excretion and alterations of protein expression of eNOS and iNOS in SHR, and demonstrated that the attenuated effects of long term electroacupuncture involved the increase of eNOS expression, and the decline of iNOS expression, in aorta and myocardium. These findings indicated that long term electroacupuncture might prevent cardiac hypertrophy and fibrosis through modulation of NOS/NO-mediated pathway. High blood pressure stimulates both fibroblasts and cardiomyocytes to express cytokines, such as angiotensin II, and results in hypertrophy and fibrosis [2]. Reduction in systolic blood pressure could attenuate these pathological changes and improve the prognosis of patients with cardiac malfunction.

Our preliminary experiments showed that compared to other acupuncture points, such as Yanglingquan (GB34), Hegu (LI4), Quchi (LI11), and Neiguan (PC6), electroacupuncture stimulation at DU20 and ST36 exerts a more apparent antihypertensive effect. Stimulation at ST36 alone was also reported to prevent the progression of hypertension and diminish the cardiovascular remodeling in SHR [20]. However, the study regarding the ameliorating effect of electroacupuncture at both acupoints DU20 and ST36 on cardiac hypertrophy and fibrosis in hypertensive rats is limited. In addition, in a preliminary study, we evaluated the effect of electroacupuncture on the mean arterial pressure in SHR rats at 16 weeks of stimulating at both DU20 and ST36 acupoints or only one of the two. The result showed a more pronounced effect of electroacupuncture when applied at both acupoints than that at any single one. Since the objective of this study was to explore the mechanisms whereby electroacupuncture attenuates cardiovascular remodeling, we thus chose a most effective application manner, that is, stimulation at both acupoints. In the present study, electroacupuncture stimulation at DU20 combined with ST36 significantly relieved hypertension in SHR as well as recovered cardiovascular remodeling. However, whether the MAP decrease is causative rather than epiphenomenal in the therapeutic effect of long term electroacupuncture at acupoints DU20 and ST36 on cardiovascular protection requires further study.

LV hypertrophy and aortic thickening are two common pathological manifestations in hypertensive animals. Previous researches have proved that Ang II in vivo, via AT1 receptor, directly induces cardiac myocyte hypertrophy and vascular growth, and probably fibroblast proliferation and subsequent fibrosis as well, indicating the key role of Ang II in the development of pathological cardiac remodeling and vascular thickening [21]. As shown in our study, there was a significantly greater increase in the LVAW, LVPW, HW/BW ratio, collagen deposition and aortic wall thickness in SHR than in Wistar. And the levels of AngII and AT1R in myocardium and ascending aorta were higher in SHR than in Wistar. Treatment with electroacupuncture stimulation at acupoints DU20 and ST36 for 8 weeks significantly decreased the cardiac hypertrophy and fibrosis index accompanied by a lower level of Ang II and AT1R in myocardium and aorta. These results suggest that electroacupuncture at acupoints DU20 and ST36 can diminish the remodeling of the heart and aorta in SHR via local renin-angiotensin system depression.

Existence of an AT1R-ET-1-ETAR pathway in hypertension pathogenesis is well recognized, that is, an increased interaction of Ang II with AT1R stimulates the release of ET-1 that enhances the binding of ET-1 with ETAR, leading to vasoconstriction [22,23]. Previous studies suggested that ET operates downstream of the TGFβ/Ang II system to drive fibroblast activation and fibrosis [6]. Manipulation of ET-1 system is thus pivotal for attenuating high blood pressure and pathological remodeling of the heart. Our study suggested an implication of ET-1-ETAR pathway in alleviating MAP and cardiac remodeling by electroacupuncture at DU20 and ST36, which suppressed the expression of ETAR and reduced the content of ET-1 in SHR. The signaling pathway that mediates the effect of electroacupuncture on DU20 or ST36 alone is at present unknown, and if stimulation at both acupoints confers synergistic effects and additional regulatory effects waits for further study.

Previous studies have indicated that acupuncture on DU20 or ST36 alone could restore NO in the peripheral blood of SHR [10,20]. In our study, electroacupuncture stimulation at both DU20 and ST36 resulted in a significant increase in plasma NO, which suggested that the effect of the electroacupuncture on blood pressure may be related to an enhanced biosynthesis or release of NO. The increase in plasma NO following the electroacupuncture treatment may also be related to the remodeling attenuation, but this is yet to be confirmed by further investigations. Altered NOS expression has been observed in both human and animal models of heart failure [24-26]. In the present study, electroacupuncture at both DU20 and ST36 improved the decreases in cardiac and aortic eNOS protein expression, while inhibited enhancement of cardiac and aortic iNOS protein expression, in SHR group. These results indicated that long term electroacupuncture may ameliorate cardiac and vascular remodeling by promoting eNOS and inhibiting iNOS protein expression in myocardial and aortic tissues.

An interesting question arises then: why electronacupuncture had no effect on Wistar rats in terms of the parameters concerned in the present study? We speculate that electronacupuncture has a potential to correct the disordered signaling, e.g., the AT1R-AT1R-ET-1-ETAR and NOS/NO pathway in SHR in the present case, but has no influence on a normal signaling, such as the AT1R-AT1R-ET-1-ETAR and NOS/NO pathway in Wistar rats. This may be the advantage of electronacupuncture over other strategy.

## Conclusions

The present study showed that long term electroacupuncture stimulation at DU20 and ST36 can significantly relieve the increased MAP not only by the inhibiting production of Ang II and ET-1 but also by the promoting NO production, and the decreased AT1R, ETAR, iNOS expression and increased eNOS expression in the cardiac and aortic tissues may be involved in the protective effect of electroacupuncture on myocardial and aortic remodeling.

## Abbreviations

Ang II: Angiotensin II; AT1R: Angiotensin II type 1 receptor; EF: Ejection fraction; ET-1: Endothelin-1; ETAR: Endothelin-1 type A receptor; FS: Fractional shortening; LVAW: Left ventricular anterior wall; LVID: Left ventricular internal diameter; LVPW: Left ventricular posterior wall; MAP: Mean arterial pressure; NO: Nitric oxide; NOS: Nitric oxide synthase; SHR: Spontaneously hypertensive rats.

## Competing interests

The authors declare that there are no financial competing interests.

## Authors’ contributions

Conception and design: HJY and LZG. Acquisition of data: HZJ, LQ, TGH, WXH, PCS and HK. Analysis and interpretation of data: ZCM, PCS, YL, BY, ZYY and WCS. Drafting the manuscript: LQ and ZCM. All authors read and approved the final manuscript.

## Pre-publication history

The pre-publication history for this paper can be accessed here:

http://www.biomedcentral.com/1472-6882/14/118/prepub
